# Hemispheric asymmetries in rodent models of neurological diseases: a systematic review

**DOI:** 10.3389/fnint.2026.1843068

**Published:** 2026-08-06

**Authors:** Maximilian Schuessler, Annakarina Mundorf

**Affiliations:** 1 Institute for Diagnostic and Interventional Radiology, Neuroradiology and Nuclear Medicine, University Hospital Knappschaftskrankenhaus Bochum, Ruhr University Bochum, Bochum, Germany; 2 Institute for Systems Medicine, Department of Human Medicine, MSH Medical School Hamburg, Hamburg, Germany

**Keywords:** Alzheimer’s disease, brain injury, epilepsy, inter-individual differences, lateralization, lesion, Parkinson’s disease, stroke

## Abstract

**Introduction:**

Hemispheric asymmetries are a core organizational principle of the vertebrate brain, but they are still not fully integrated into the study of preclinical models of neurological disease. Although many rodent models naturally include lateralization through unilateral interventions, hemispheric differences are not consistently examined or systematically reported.

**Methods:**

A systematic review was conducted following PRISMA guidelines, combining evidence from 48 studies on hemispheric asymmetries in rodent models of neurological conditions, including mild traumatic brain injury, epilepsy, stroke, Alzheimer’s, and Parkinson’s disease.

**Results:**

Across these models, disease processes cause strong lateralized effects on behavior, neurochemistry, and regional metabolism. In epilepsy models specifically, hemispheric asymmetries depend on the model: early seizure spread and network excitability can be lateralized, while other paradigms show more symmetric responses, emphasizing the role of baseline circuit asymmetries and genetic background. Similar lateralized effects are seen in stroke and Parkinson’s models, reflecting clinical phenomena such as lesion-side-dependent outcomes and unilateral symptom onset.

**Discussion:**

Models focusing on intrinsic hemispheric asymmetry or interhemispheric/bilateral response highlight existing differential sensitivity to experimental manipulations that may predispose the system to asymmetric outcomes. Nonetheless, variability and dynamic shifts in hemispheric organization are rarely taken into account. Incorporating hemispheric analysis systematically into experimental design and data interpretation could enhance the sensitivity, clarity, and clinical relevance of rodent models of neurological disease.

## 1 Introduction

Hemispheric asymmetries in the vertebrate brain refer to structural and functional differences between the left and right cerebral hemispheres and represent a fundamental organizational principle of neural systems (Güntürkün et al., 2020; Ocklenburg and Güntürkün, 2024). These asymmetries increase processing efficiency, reduce redundancy, and support parallel processing of information (Vallortigara and Rogers, 2020; Ocklenburg and Mundorf, 2022). In the context of neurological disease research, hemispheric asymmetries are particularly relevant because they can influence both experimental outcomes and disease-related phenotypes (Lubben et al., 2021; Ocklenburg et al., 2024; Mundorf et al., 2026). These changes are sometimes called “lateralized effects”; however, this term is used in different ways across studies, often without clear distinction between intrinsic brain asymmetry, disease-related changes, and effects arising from experimental design.

In this review, we use the term lateralized effects to describe structural, molecular, physiological, or behavioral differences between the left and right hemispheres. Importantly, not all lateralized findings reflect the same underlying biological phenomenon. While some asymmetries arise from intrinsic hemispheric specialization or differential susceptibility of the two hemispheres, others represent the direct consequence of unilateral experimental manipulations. Because many neural pathways, particularly sensorimotor circuits, are predominantly crossed, unilateral lesions or interventions are expected to generate ipsilateral-versus-contralateral differences that do not necessarily indicate inherent hemispheric specialization. Conversely, unilateral manipulations may also evoke bilateral or contralateral responses through interhemispheric communication and compensatory mechanisms. To distinguish these conceptually different phenomena, the studies included in this review are classified into three categories: (i) intrinsic or pre-existing hemispheric asymmetries, including asymmetries reflecting inherent or pre-configured hemispheric organization and/or differential sensitivity to left vs. right manipulations; (ii) asymmetric outcomes following unilateral manipulations, in which hemispheric differences primarily reflect the direct consequences of an asymmetric experimental intervention; (iii) interhemispheric or bilateral responses, in which unilateral perturbations evoke contralateral or bilateral adaptations that provide evidence for interhemispheric communication, compensatory plasticity, or network-wide propagation of disease effects. This framework provides a clearer basis for interpreting lateralized findings across neurological rodent models and distinguishes inherent hemispheric organization from secondary consequences of asymmetric experimental paradigms.

Beyond experimentally induced asymmetries, intrinsic hemispheric differences are present in both healthy brains and neurological disease (Mundorf and Ocklenburg, 2021). Studies in humans and rodents show consistent structural, functional, and neurochemical differences between hemispheres, although their expression depends on brain region and behavioral context (Budilin et al., 2008; Ocklenburg et al., 2014, 2022; Papp et al., 2019; Stanković, 2021; Mundorf et al., 2025a). These asymmetries are embedded in distributed neural networks and can interact with interhemispheric connectivity to shape disease expression and progression (Carson, 2020; Bonkhoff et al., 2021; Peterson et al., 2025). Among the most investigated brain regions in terms of structural and functional asymmetries in rodents and humans are the hippocampus (Nemati et al., 2023) and the amygdala (Ocklenburg et al., 2022). Interestingly, intrinsic asymmetries are altered in psychiatric and neurological disorders (Lubben et al., 2021; Mundorf and Ocklenburg, 2021; Mundorf et al., 2024, 2025a; Ocklenburg et al., 2024; Packheiser et al., 2025). Parkinson’s disease, Alzheimer’s disease, stroke, and epilepsy frequently show lateralized pathology or symptom expression, although the degree and direction of asymmetry vary across individuals and disease stages (Wachinger et al., 2016; Balestrini et al., 2021; Li et al., 2021; Roe et al., 2021; Steinbach et al., 2021; Mundorf et al., 2025b). This variability highlights the translational relevance of hemispheric organization but also introduces methodological challenges for both preclinical and clinical research.

Despite this, hemispheric lateralization is still rarely assessed systematically. In many studies, data from both hemispheres are pooled, and unilateral experimental manipulations are analyzed without explicit reporting or balancing of the affected hemisphere (Mundorf and Ocklenburg, 2025a,b). This is problematic because, as seen in both rodents and humans, lateralization shows substantial inter-individual variability, including differences in baseline hemispheric dominance across behavioral and structural measures (Sacher et al., 2020; Manns et al., 2021; Elkind et al., 2023; Gerrits, 2024; Ocklenburg et al., 2024). As a result, important sources of biological variation may be overlooked.

Taken together, two key issues remain underexplored in preclinical research: the magnitude and variability of disease-related hemispheric asymmetries, and the role of interhemispheric interactions in shaping disease mechanisms and progression. Addressing these issues is essential for improving the interpretability, reproducibility, and translational value of experimental studies. In the following sections, we review hemispheric asymmetries in rodent models of neurological disease, discuss common methodological limitations such as missing hemisphere reporting and lack of counterbalancing in unilateral designs, and highlight the importance of accounting for inter-individual differences in baseline lateralization.

## 2 Methods

### 2.1 Search strategy and information sources

A systematic literature search was conducted to identify preclinical studies investigating hemispheric asymmetries in rodent models of neurological diseases in line with PRISMA guidelines (Page et al., 2021). The search was performed in PubMed and EBSCO (including the following databases: APA PsycInfo, APA PsycArticles, CINAHL Complete, MEDLINE Complete, and Psychology and Behavioral Sciences Collection) from inception to January 13*^th^* 2026. The following search string was used: (“hemispheric asymmetry” OR “hemispheric asymmetries” OR “hemispheric difference*” OR “functional lateralization” OR “functional lateralization” OR “brain lateralization” OR “brain lateralization” OR “left-right difference”) AND (mouse OR mice OR rat OR rats OR rodent*) AND (stroke OR ischemia OR ischemic OR “middle cerebral artery occlusion” OR MCAO OR epilepsy OR epileptic OR seizure* OR glioma OR glioblastoma OR “brain tumor” OR “brain tumor” OR Parkinson* OR “dopaminergic neuron*” OR 6-OHDA OR MPTP OR Alzheimer* OR amyloid OR tau OR “traumatic brain injury” OR TBI OR “spinal cord injury” OR SCI OR demyelination OR “multiple sclerosis” OR EAE).

### 2.2 Eligibility criteria

Studies were included if they reported original research in rodents (mice, rats, or other rodent species) modeling neurological diseases, including stroke, epilepsy, glioma, Parkinson’s disease, Alzheimer’s disease, traumatic brain injury, spinal cord injury, or demyelination/multiple sclerosis. Eligible studies needed to assess hemispheric asymmetry, functional lateralization, or left-right differences in the brain. Reviews, conference abstracts, case reports, and studies not reporting hemispheric or lateralized outcomes were excluded. Only articles published in English were considered.

### 2.3 Study selection, data extraction, and synthesis

Titles and abstracts were first screened, followed by full-text review of studies that met the inclusion criteria or had unclear eligibility. The selection process was documented using a PRISMA flow diagram, detailing records identified, screened, excluded, and included. Data were extracted using a standardized form capturing study characteristics (authors, year, rodent species), modeled neurological disease, experimental interventions, methods for assessing hemispheric asymmetry, and main lateralization outcomes. Given the heterogeneity of rodent species, disease models, and outcome measures, results were synthesized narratively and grouped by disease model, summarizing left-right differences and highlighting consistent patterns as well as major discrepancies across studies.

## 3 Results

### 3.1 Study selection

**FIGURE 1:**
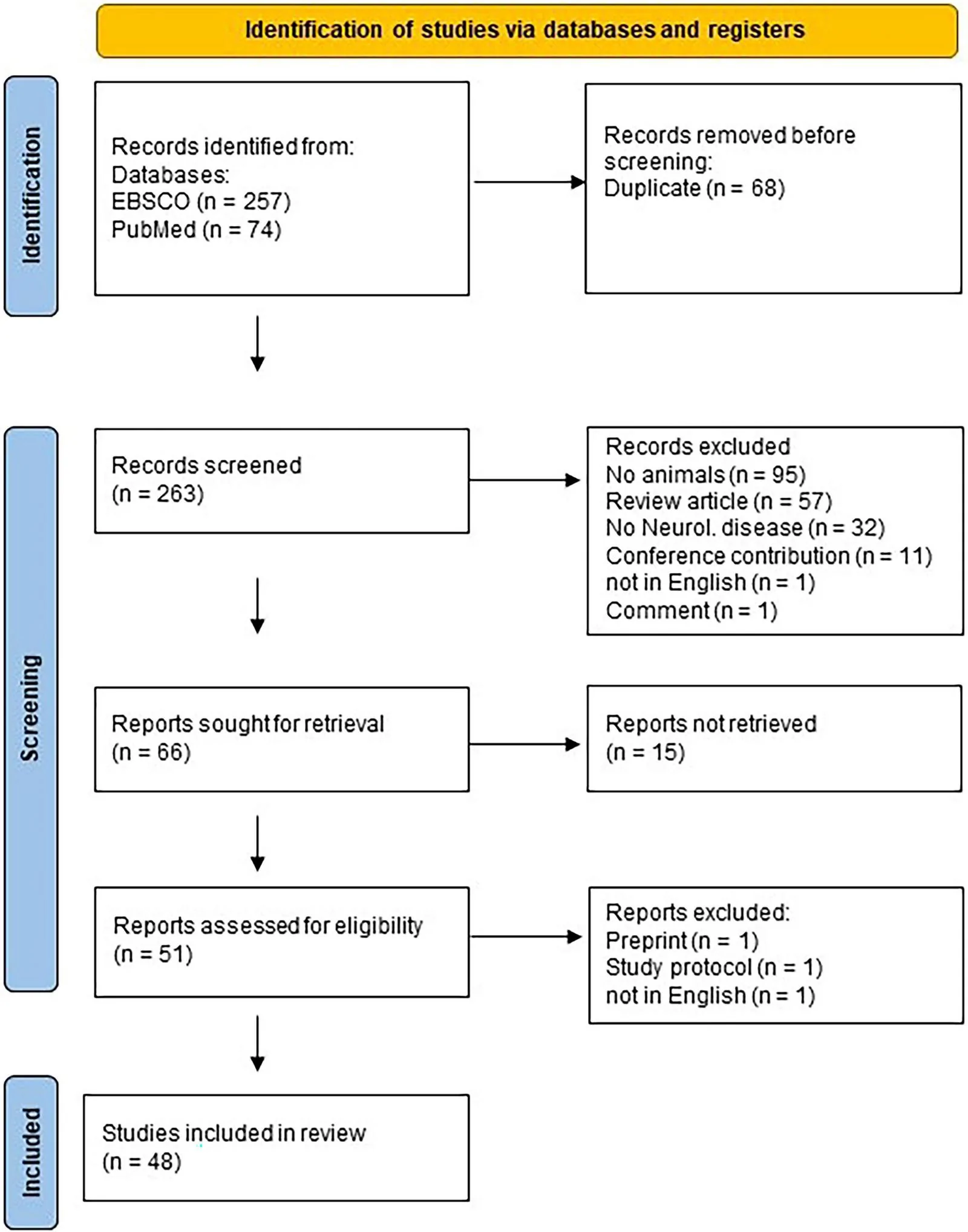
PRISMA flow diagram illustrating the literature search, screening, and selection process, adapted from Page et al. (2021).

The initial database search yielded 331 records. After removal of duplicates, 263 records remained for title and abstract screening. Following this screening, 66 articles were sought for retrieval, and 51 full-text articles were assessed for eligibility. Of these, 48 studies met the inclusion criteria and were included in the review, while three were excluded: one was a preprint, one was a study protocol, and the other was not in English. The study selection process is summarized in a PRISMA flow diagram (Figure 1).

### 3.2 Included animal studies of neurological diseases

The included animal studies investigating hemispheric asymmetries in models of neurological diseases are presented below, organized by model type, including models of mild traumatic brain injury, seizures and epilepsy, stroke, Alzheimer’s disease, Parkinson’s disease, and other relevant conditions (one lesion and one tumor study). Where reported in the original study, the strain and age of animals were noted. Among the included neurological models, the majority of studies investigated asymmetric outcomes following unilateral manipulation, mainly using unilateral lesion paradigms such as 6-OHDA models. These studies consistently demonstrated ipsilateral/contralateral differences in behavioral performance, neuronal activity, metabolism, or molecular responses after unilateral damage (Table 1). However, these approaches primarily demonstrate consequences of experimentally imposed asymmetry and provide limited information about pre-existing hemispheric vulnerability.

A smaller number of studies investigated intrinsic hemispheric asymmetries or used models allowing assessment of individual lateralization. These studies, including developmental and genetic models, suggest that hemispheric differences may precede pathology and potentially influence regional vulnerability. Furthermore, some studies observed normalization of hemispheric differences during functional recovery, indicating that compensatory mechanisms may contribute to restoring interhemispheric balance after unilateral injury (Table 1).

**TABLE 1:** Summary of the included studies, organized by disease model, species/strain, type of experimental induction, type of lateralized effect (intrinsic hemispheric asymmetry, asymmetric outcome following unilateral manipulation, or interhemispheric/bilateral response), and the principal structural and functional findings.

Disease model	References	Species/ strain	Type of induction	Type of lateralized effect	Structural findings	Functional findings	Behavioral findings	Examples of converging findings in humans
Mild traumatic brain injury	Griesbach et al., 2002	Sprague-Dawley rats, P19, males	lateral FPI	Interhemispheric/ bilateral response	–	•↑ Contralateral BDNF mRNA (hippocampus, occipital cortex) •↑ Bilateral hippocampal BDNF protein (7 days), later ipsilateral only (14 days) • Ipsilateral ↑ trkB and synapsin I; contralateral ↓ trkB and synapsin I • Compensatory interhemispheric neuroplasticity	– –	• TBI can result in abnormal hemispheric brain volume asymmetry, including region-specific enlargement and atrophy (Ross et al., 2023) • Altered limbic volume asymmetry after mTBI is associated with symptom persistence (Vanier et al., 2024) • MTBI reduces hemispheric white matter symmetry in specific tracts (Vakhtin et al., 2020)
	Giza et al., 2006	Sprague-Dawley rats, P19, males	Lateral FPI	Asymmetric outcome following unilateral manipulation	–	•↓ Ipsilateral hippocampal NR2A protein • Bilateral cortical NR2A reduction •No hemispheric differences in cortical NR1/ NR2B or mRNA expression		
	Murugan et al., 2016	Sprague-Dawley rats, P25-P26, males	developmental lateral FPI, left hemisphere	Interhemispheric/ bilateral response	• Ipsilateral neuronal loss (hippocampus)	• Altered whisker-evoked CBF responses (sensorimotor cortex) •↓ Positive CBF response, ↑ negative CBF response after mTBI • Interhemispheric asymmetry of neurovascular activity • Kaempferol restored bilateral CBF symmetry	• Persistent contralateral sensorimotor deficits • Impaired whisker stimulation-induced motor response •↑ sensorimotor behavior after treatment	
	Divani et al., 2015	Wistar rats, males	Unilateral blast-induced TBI (shock wave lithotripsy; right frontal cortex)	Asymmetric outcome following unilateral manipulation	• Histological injury severity (OHS score) correlates with tissue damage extent	• Mild hemispheric differences in cerebral perfusion (DSA contrast peak values) • Transient interhemispheric perfusion asymmetry (early time points) • No sustained hemispheric divergence at 7 days	•↓ motor coordination (beam walking, rotarod) •↑ anxiety-like behavior (elevated plus maze) • Neurological deficits (modified Garcia score) • Behavioral severity correlates with injury burden	
	Schoettle et al., 1990	Wistar rats, males	Right hemispheric percussive TBI	Asymmetric outcome following unilateral manipulation	• Stable contralateral hemisphere water content vs. sham (no edema) • Progressive ipsilateral brain edema (R–L difference in brain water)	• Labeled PMN accumulation index reflects ipsilateral injury + intravascular blood + inflammatory migration • Early PMN signal partly reflects hemorrhage/ blood volume (0–2 h) • True PMN infiltration evident at 4–8 h after correction for blood volume (BVI) • Hemispheric BVI used to separate vascular vs. inflammatory contribution	–	
	Schurman et al., 2017	C57BL/ 6J mice, males	Left or right lateral FPI (parietal cortex)	Intrinsic hemispheric asymmetry (left vs. right manipulation effects)	• Similar lesion volumes (left vs. right injury)	• Similar neuroinflammatory response patterns • Ipsilateral > contralateral microglial/ astrocytic activation (both left and right injury)	• Comparable motor deficits after left vs. right injury (righting reflex, rotarod) • Right injury: slight delay in acquisition (MWM fixed platform) • Both injuries: probe trial deficits (MWM fixed platform) • Left injury: mild deficits in reversal learning (MWM) • No hemispheric differences in motor outcomes	
	Tashlykov et al., 2007	ICR mice, males	Closed head weight-drop (right-sided impact; minimal TBI)	Interhemispheric/ bilateral response	•↑ TUNEL-positive cells in left and right cortex •↑ TUNEL-positive cells in left and right hippocampus •↑ Silver-stained degeneration in left and right hemispheres • Regional vulnerability in bilateral ACC and CA3	–	–	
Seizure and epilepsy	Vinogradova and Shatskova, 2012	Wistar and WAG/ Rij rats, males	Audiogenic kindling model of generalized tonic-clonic seizures	Intrinsic hemispheric asymmetry	–	•Asymmetric brainstem-to-forebrain seizure propagation; • ipsilateral cortical recruitment; • No population-level bias in lateralization	• Individualized running direction (left/ right) • Right-runners show ↓ cortical seizure incidence (Wistar) • Strain-dependent differences in seizure expression	•Generalized epileptiform discharges in idiopathic generalized epilepsy show consistent hemispheric asymmetry without a left-right bias, indicating lateralized network involvement (Pizarro et al., 2025) • Mesial temporal lobe epilepsy shows altered functional connectivity and reduced interhemispheric asymmetry, with distinct patterns in left- and right-sided epilepsy (Zhao et al., 2022)
	Tchekalarova et al., 2016	Spontaneously hypertensive rats and Wistar rats, males	Kainate model of temporal lobe epilepsy	Intrinsic hemispheric asymmetry	–	•↑ Baseline delta power in left frontal cortex in SHRs •↓ Gamma/ HF power in left frontal and bilateral parietal cortex in SHRs (baseline) • Acute phase: ↓ fast oscillations (gamma/ HF) bilaterally in SHRs • Latent phase: no significant differences • Chronic phase: ↑ HF power in frontal/ parietal cortex (no lateralization); ↓ theta/ alpha/ beta in left parietal cortex	–	
	Mesquita et al., 2011	Wistar rats, males	Electrical stimulation (right basolateral amygdala; periodic vs non-periodic inter-pulse interval; with and without pentylenetetrazole-induced seizure	Interhemispheric response	–	• No hemispheric differences in during electrical stimulation alone • During pentylenetetrazole infusion: periodic stimulation ↑ ipsilateral temporal and amygdaloid network recruitment • Non-periodic stimulation ↓ ipsilateral recruitment and ↓ interhemispheric differences • Seizure network synchronization versus desynchronization depended on stimulation temporal structure under epileptogenic state	–	
	Myslobodsky and Rosen, 1979	Rats (not specified)	Pentamethyle- netetrazol-activated seizure	Intrinsic hemispheric asymmetry (nigrostriatal dopamine system-related functional lateralization)	–	• Wave–spike activity: ↑ in one hemisphere/ ↓ in contralateral hemisphere • Interhemispheric asymmetry of epileptiform bursts	• Circling toward hemisphere with ↓ wave–spike activity • Rotation direction matches EEG asymmetry	
	Fan et al., 2008	Sprague-Dawley rats	Prenatal methylazoxymethanol and thalidomide exposure	Interhemispheric/ developmental network-mediated effects	• Early ventricular size asymmetry • Early ventricular size asymmetry • Transient hemispheric volume imbalance during development • Altered brain water homeostasis • No persistent hemispheric asymmetry in adulthood	• Long-term hippocampal network reorganization without clear left–right dominance	–	
	Yorulmaz et al., 2013	Wistar rats, not specified	Zinc supplementation ± pentylenetetrazole- induced seizures	Interhemispheric asymmetry (metabolic/ ionic distribution differences)	• Control: Zn left > right; Na right > left (brainstem-related); Mg/ Cu no stable L/ R pattern • Pentylene-tetrazole: Na left > right; Mg right > left; Zn differences present without consistent direction • Zinc: Zn left > right; Na left > right; Mg right > left; Cu right > cerebellum/ brainstem • Zinc + pentylene-tetrazole: Mg left > right; Zn/ Na/ Cu hemispheric differences present but mixed direction	• Blood-brain barrier permeability ↑ and asymmetric across hemispheres in all conditions • Zinc and pentylene-tetrazole modulate magnitude and direction of interhemispheric ionic imbalance	–	
	Cain et al., 1989	Hooded rats, males	Amygdala kindling (left vs. right basolateral amygdala; within-subject threshold screening)	Intrinsic hemispheric asymmetry	No difference between left vs right kindling	–	–	
	Gelbard and Applegate, 1994	Sprague-Dawley rats, males	Amygdala and piriform cortex kindling (left vs. right hemisphere; within-subject threshold screening)	Intrinsic hemispheric asymmetry	No hemispheric differences in D2 receptor mRNA expression (accumbens, striatum)	–	–	
Stroke	Robinson and Coyle, 1980	Sprague-Dawley rats, males	Right vs. left MCA ligation	Asymmetric outcome following unilateral manipulation	Similar lesion appearance (fronto-parietal cortical depression)	• Right hemisphere infarction: ↓ catecholamines in cortical and subcortical regions • Left hemisphere infarction: no consistent neurochemical changes vs. control	• Right hemisphere infarction: ↑ activity open field and running wheel; ↑ locomotion across measures • Left hemisphere infarction: no change vs. sham in activity, exploration, or locomotion	• Stroke lateralization is associated with asymmetric vascular anatomy and cerebral hemodynamics, predicting left-hemispheric ischemic stroke (Abdelnaby et al., 2023) • Right-hemispheric ischemic stroke is associated with higher rates of malignant brain edema and cardiovascular complications than left-hemispheric stroke (Li et al., 2021)
	Arteni et al., 2010	Wistar rats, P7	Neonatal unilateral HI (left vs. right hemisphere)	Asymmetric outcome following unilateral manipulation	↑ Ipsilateral striatal atrophy after right-HI (more pronounced in females)	–	• Right-HI ↑ locomotor activity (males) • Right-HI ↓ working memory (females) • Right-HI ↓ aversive learning • Spatial learning deficits after HI regardless of lesion side	
	Sanches et al., 2013a,b	Wistar rats	HI-P3, occlusion of the right or left carotid artery at P3	Asymmetric outcome following unilateral manipulation	• HIP3-L females: ↓ ipsilateral hemispheric volume at PND21 • HIP3-L females: ↓ corpus callosum area (↑ white matter damage) at PND21 and PND90 • No hippocampal volume changes after HI	• Early after HI: right hemisphere ↓ Na^+^/ K^+^-ATPase activity • Females: ↓ Na^+^/ K^+^-ATPase activity ipsilateral vs. males	• Left-HI females: ↑ spatial memory deficits, ↑ inhibitory avoidance deficits • Right-HI: ↑ anxiety-like behavior • No persistent lateralized motor deficits	
	Sanches et al., 2015	Wistar rats, P3	Neonatal unilateral HI (P3 vs. P7; left vs. right carotid occlusion)	Asymmetric outcome following unilateral manipulation	• HIP3-L females: ↓ total hemispheric volume • HIP7-R females: ↓ hemispheric volume vs. HIP7-L • HIP7-R males and females: ↓ hippocampal volume vs HIP7-L	–	• HIP3-L females: ↑ cognitive deficits • Behavioral impairments depended on sex, age, and injured hemisphere • HIP7: ↑ motor and cognitive deficits	
	Besselmann et al., 2001	Wistar rats, males	Right unilateral MCA occlusion (intraluminal thread model; transient ischemia/ reperfusion)	Asymmetric outcome following unilateral manipulation	No difference in MCA vascular branching	• Ipsilateral perfusion ↓ (∼30% of contralateral side) by PWI • Ipsilateral diffusion restriction by DWI indicating ischemic tissue changes	–	
	Novitzky et al., 2016	C57Bl/ 6 mice, males	Left unilateral MCA occlusion	Asymmetric outcome following unilateral manipulation	•↑ Infarct volume and brain edema in the injured hemisphere • Delayed bevacizumab treatment: ↓ brain edema and stroke volume	•↓ Perfusion in the injured hemisphere • Bevacizumab treatment: ↓ neurological deficits	–	
	Michalski et al., 2018	Sv129/ B6 wild-type and triple-transgenic Alzheimer-model mice, 3- and 12-month-old	Right-sided permanent MCA occlusion	Asymmetric outcome following unilateral manipulation	Right hemisphere (ischemic): ↑ MBP vs. left hemisphere (contralateral) • Right hemisphere (ischemic): ↑ OSP vs. left hemisphere • Right hemisphere (ischemic): ↑ CNPase, ↑ MBP, ↓ NG2 vs. contralateral hemisphere • Within right hemisphere: ischemic core > border zone > peri-infarct cortex gradient	• Neurovascular unit remodeling localized to right (ischemic) vs. left hemisphere • Myelin/ oligodendrocyte marker changes predominantly restricted to right hemisphere	–	
	Zhou et al., 1989	Rats, not specified	Left carotid occlusion and hypotension	Asymmetric outcome following unilateral manipulation	–	• EEG amplitude ↓ in left (ipsilateral to carotid occlusion) vs. right hemisphere • Hemispheric asymmetry during progressive hemorrhagic hypotension • MAP threshold for EEG asymmetry modulation depends on anesthetic condition (no hemispheric specificity)	–	
	Volkov et al., 2011	Rats (not specified), males	Focal ischemic stroke in sensorimotor cortex (dominant vs. non-dominant hemisphere)	Intrinsic hemispheric asymmetry (paw preference)	–	• Functional recovery differs depending on dominance: dominant hemisphere lesion → slower recovery than non-dominant hemisphere lesion	• Forelimb function (cylinder test, whisker test, swimming): no left vs. right lesion difference • Dominant hemisphere stroke: impaired recovery of forelimb function vs. non-dominant hemisphere stroke • 8-week follow-up: persistent recovery asymmetry based on dominance	
Alzheimer’s disease	Morrissey et al., 2022	AppNL-G-F mice	Genetic AD model	Interhemispheric or bilateral responses	–	• Loss of normal hemispheric asymmetry in hippocampal functional connectivity	–	• Reduction in cortical hemispheric asymmetry accelerated (Roe et al., 2021) • Asymmetric tau distribution (70% of patients) associated with earlier disease onset and more severe pathology (Lu et al., 2023) • Left-right asymmetry of tau pathology linked to amyloid-beta laterality and differential cognitive decline, independent of interhemispheric connectivity (Anijärv et al., 2025) • Trend toward stronger right-hand preference, although limited (Mundorf et al., 2025b)
	Biechele et al., 2021	AppNL-G-F mice	Genetic AD model	Intrinsic hemispheric asymmetry	–	• Right amygdala–entorhinal/ hippocampal microglial activation (TSPO-PET ↑) associated with preserved/ improved cognitive performance • No significant left hemispheric associations	•↑ Right amygdala TSPO expression associated with ↑ spatial learning performance • Right entorhinal/ piriform cortex and amygdala TSPO signal correlated with Morris water maze performance	
	Sacher et al., 2020	APP/ PS1, PS2APP, APP-SL70, AppNL-G-F, and APPswe mice	Genetic AD model	Intrinsic hemispheric asymmetry	• Aβ deposition showed hemispheric asymmetry in ≥30% of mice • Single hemispheres showed ↑ variability in Aβ burden due to asymmetry	• Aβ PET asymmetry correlated with TSPO-PET asymmetry (microglial activation) • Hemispheric predominance of amyloid pathology associated with neuroinflammatory asymmetry	–	
	Chen et al., 2019	APP/ PS1 and wildtype mice	Genetic AD model	Interhemispheric or bilateral responses	–	•↑ Gamma-band LFP synchronization between left & right secondary motor cortex •↑ Interhemispheric coupling and ↓ hemispheric asymmetry	–	
	O’Riordan et al., 2018	Wistar and Lister hooded rats, males	Amyloid-β–associated AD model & bilateral optogenetic stimulation of hippocampal Schaffer collateral pathways to assess LTD	Intrinsic hemispheric asymmetry	–	• No LTD asymmetry between left and right hippocampus under control conditions • Aβ selectively ↑ LTD induction in left hippocampus • Aβ-induced left-sided LTD ↑ is mGluR5-dependent	–	
Parkinson’s disease	Day et al., 2002	Sprague-Dawley rats, males	Unilateral 6-OHDA lesions of the medial forebrain bundle (left and right counter-balanced)	Asymmetric outcomes following unilateral manipulation	–	• Lesioned hemisphere: ↓ CRH and neurotensin mRNA expression in central amygdala% oval nucleus of bed nucleus of stria terminalis • Ipsilateral striatum: ↑ neurotensin and enkephalin mRNA expression • No changes in CRH expression in cortex, hypothalamus, or ventral bed nucleus of stria terminalis	–	• Motor symptoms show a bias toward the dominant side (van der Hoorn et al., 2012) • Left-hemispheric dopaminergic loss is associated with cognitive impairment, whereas right-hemispheric loss is associated with greater motor severity (Fiorenzato et al., 2021) • Right-sided symptom onset is associated with more severe white matter damage than left-sided onset (Zhu et al., 2023)
	Neveu et al., 1992	C3H mice, females	Left or right 6-OHDA lesions of SNr	Asymmetric outcomes following unilateral manipulation	—–	• Left SNr lesion: ↓ splenic lymphocyte mitogenesis • Right SNr lesion: ↑ splenic lymphocyte mitogenesis • Natural killer cell activity unaffected by lesions	–	
	Carlson et al., 1999	Sprague-Dawley rats, males	Pedunculopontine nucleus of 6-OHDA-lesioned (right-sided based on stereotaxic coordinates)	Asymmetric outcomes following unilateral manipulation	–	• Ipsilateral globus & pedunculopontine nucleus pallidus: ↑ glucose metabolism • Ipsilateral • primary motor, sensory, and auditory cortex and subthalamic nucleus: ↓ glucose metabolism	Apomorphine-induced rotations contralateral to the lesion (leftward rotations)	
	Dwyer et al., 2021	C57Bl6 mice, males	Systemic neurotoxin exposure (intraperitoneal injections)	Intrinsic hemispheric asymmetry	•↓ SNc hemispheric size ratio (left vs. right) •↓ Asymmetry most pronounced after 6 months	–	–	
	Cunha et al., 2020	Wistar-Han rats, males	Unilateral 6-OHDA lesion of left or right NAc	Asymmetric outcomes following unilateral manipulation	–	–	• Right lesion: ↓ of lever pressing after contingency degradation → impaired goal-directed behavioral flexibility • Left lesion: ↑ premature responding during delayed signal task →↑ impulsivity • No differences in rewards consumption, anxiety/ depressive-like behavior, or motor performance	
	Farré et al., 2015	Wistar rats, males	Unilateral 6-OHDA lesion	Asymmetric outcomes following unilateral manipulation	–	Left striatum: •↑ D1 receptor agonist affinity (baseline dopamine receptor imbalance) • D3 receptor activation during dyskinesia: ↑ right striatal D1 receptor dopamine sensitivity, ↓ left-right imbalance	↓ Use of contralateral left forepaw in cylinder test	
	Rohlfs et al., 1997	Sprague-Dawley rats, female	Unilateral 6-OHDA lesion in right SNr	Asymmetric outcomes following unilateral manipulation	• Right SNc: near-complete loss of dopaminergic neurons after 6-OHDA lesion	• Ipsilateral SNr: ↓ spontaneous firing rate and ↑ bursting activity • Contralateral SNr: ↑ spontaneous firing rate compared with controls	–	
	Ishida et al., 1998a,b	Wistar rats, female	Unilateral left-sided 6-OHDA lesion of the medial forebrain bundle; Methamphetamine challenge inducing neuronal activation	Asymmetric outcomes following unilateral manipulation	–	• Contralateral medial striatum: ↑ methamphetamine-induced Fos expression • Ipsilateral SNr: ↑ methamphetamine-induced Fos expression •↑ SNr Fos expression is ↓ by D1/ D2 dopamine receptor and NMDA receptor antagonists	• Methamphetamine-induced rotational behavior after lesion • Dopamine D1/ D2 and NMDA receptor antagonists ↓ methamphetamine-induced rotation	
	Sullivan et al., 1994	Sprague-Dawley rats, males	Unilateral left- or right-sided 6-OHDA lesion of SNr	Asymmetric outcomes following unilateral manipulation	–	–	•↑ Edge-oriented behavior with intact striatum positioned contralateral to edge • Higher 6-OHDA dose: ↑ magnitude of orientational asymmetry • No population-level left/ right hemispheric difference	
	Johnson et al., 1999	Long-Evans rats, females	Unilateral 6-OHDA lesion of the dominant nigrostriatal dopamine pathway (assess with amphetamine-induced rotational behavior)	Intrinsic hemispheric asymmetry	–	–	• Turning: ↑ drug-induced and spontaneous turning • Rearing: ↓ duration of individual rears; ↑ no-wall-contact rears; no change in total rearing number or time • Paw use: ↓ contralateral paw use and ↓ simultaneous paw use	
	Kozlowski and Marshall, 1983	Sprague-Dawley rats, males	Unilateral left-sided 6-OHDA injection into ventral tegmental area	Asymmetric outcomes following unilateral manipulation	–	• 3 days: ipsilateral injured side: ↓ metabolism in dopaminergic target regions; ↑ metabolism in striatal output regions • 6 weeks: persistent asymmetry in non-recovered animals; normalization in recovered animals	3 days after lesion: severe impairment in contralateral tactile localization	
	Sullivan et al., 2014	Sprague-Dawley rats	Unilateral left or right vmPFC 6-OHDA lesion	Asymmetric outcomes following unilateral manipulation	No specific left/ right difference in dopamine loss	–	• Left vmPFC lesion: ↓ latency to initiate burying; ↑ burying duration and ↑ burying bout frequency; ↓ sucrose consumption • Right vmPFC lesion: altered risk assessment/ initial predator odor investigation (males: ↓ open arm time; females: ↑ open arm time) • No lesion effect on plasma ACTH	
	Papp et al., 2019	Wistar rats, males	Bilateral or unilateral (left vs. right) injections into prelimbic-PFC of dopamine agonist and antagonist	Asymmetric outcomes following unilateral manipulation	–	–	• Left PFC injection of D1 antagonist, D2 antagonist, or D3 agonist: ↓ novel object recognition at 1 h; ↑ novel object recognition at 24 h • Right PFC injection: no effect on performance	
Others	London et al., 1984	Fischer-344 rats, males	Unilateral left ibotenic acid lesion of nucleus basalis magnocellularis (ventromedial globus pallidus)	Asymmetric outcomes following unilateral manipulation	–	• 3 days after lesion: ipsilateral: ↓ glucose metabolism compared to contralateral • 28–32 days after lesion: no hemispheric difference in glucose metabolism • Receptor agonist treatment: ↑ glucose metabolism in lesioned cortex, but asymmetry remained	–	–
	Blasberg et al., 1983	Sprague-Dawley rats	Prenatal N-ethyl-N-nitrosourea exposure (single injection during gestation day 15–18)	Intrinsic hemispheric asymmetry	No hemispheric differences in blood flow related to the side of primary tumor growth	–	–	Right-hemispheric gliomas are associated with higher preoperative anxiety, whereas left-hemispheric gliomas are associated with poorer survival outcomes (Zhao et al., 2025)

6-OHDA, unilateral 6-hydroxydopamine; AD, Alzheimer’s disease; CBF, cerebral blood flow; DWI, diffusion-weighted imaging; HI, hypoxia-ischemia; FPI, fluid percussion injury; LFP, local field potential; LTD, long-term depression; MAP, mean arterial blood pressure; MCA, middle cerebral artery; NAc, nucleus accumbens; P, postnatal day; PFC, prefrontal cortex; PWI, perfusion-weighted imaging; SNr, substantia nigra; vmPFC, ventromedial prefrontal cortex. Age is indicated only for non-adult animals. If not specified, both sexes were included.

### 3.3 Models of mild traumatic brain injury

Findings in humans suffering from mild traumatic brain injury (mTBI) indicate altered intrinsic hemispheric asymmetries. For example, TBI resulted in abnormal hemispheric brain volume asymmetry, including region-specific enlargement and atrophy (Ross et al., 2023). Moreover, altered limbic volume asymmetry after mTBI is associated with symptom persistence (Vanier et al., 2024), and patients demonstrated reduced hemispheric white matter symmetry across several bilateral tracts (Vakhtin et al., 2020).

In experimental animal models of mTBI, hemispheric asymmetries are frequently reported across molecular, cellular, vascular, and behavioral domains, particularly in paradigms involving unilateral injury induction. In a lateral fluid percussion injury (FPI) model in developing Sprague-Dawley rats [postnatal day (P) 19], marked interhemispheric differences in neurotrophic signaling were observed, including a contralateral upregulation of BDNF mRNA in the hippocampus and occipital cortex, alongside inverse lateralization of synaptic plasticity markers (trkB and synapsin I: increased ipsilaterally, decreased contralaterally), indicating region-specific and directionally opposed plasticity responses across hemispheres (Griesbach et al., 2002). Similarly, in the same injury model, NMDA receptor subunit NR2A was selectively reduced in the ipsilateral hippocampus, whereas cortical reductions were bilateral, indicating region-specific differences in the balance between intra- and interhemispheric responses following injury (Giza et al., 2006).

Functional and behavioral asymmetries are also evident following mTBI. In a developmental mTBI model in Sprague-Dawley rats (P25-P26), animals exhibited persistent contralateral sensorimotor deficits in conjunction with ipsilateral neuronal loss, as well as asymmetric neurovascular responses to sensory stimulation, which could be normalized by enhancing mitochondrial calcium uptake, linking interhemispheric imbalance to metabolic dysfunction (Murugan et al., 2016). In a blast-induced TBI model in adult Wistar rats, focal right-sided injury resulted in transient hemispheric differences in cerebral perfusion, further supporting the presence of lateralized vascular responses following trauma (Divani et al., 2015). Early work in Wistar rats additionally demonstrated right-left asymmetries in polymorphonuclear leukocyte accumulation and cerebral edema formation following percussive injury, indicating that inflammatory responses may also contribute to lateralized secondary injury processes (Schoettle et al., 1990).

In a lateral FPI model in C57BL/6J mice, targeting either the left or right parietal cortex resulted in comparable lesion volumes, motor deficits, and glial activation, yet produced subtle side-dependent differences in cognitive performance, with right-sided injury leading to a slower acquisition rate in spatial learning tasks, while both hemispheres showed impairments in memory retention (Schurman et al., 2017). These findings indicate that the functional consequences of mTBI vary depending on the affected hemisphere, even in the presence of similar structural damage.

Unilateral manipulations do not necessarily result in hemisphere-specific outcomes but may also elicit bilateral (interhemispheric) responses. In a closed-head weight-drop model of minimal TBI in ICR mice, a right-sided impact resulted in bilateral apoptotic degeneration across cortical and hippocampal regions without detectable differences between hemispheres, indicating a predominantly bilateral response pattern following unilateral injury (Tashlykov et al., 2007).

Taken together, these findings demonstrate that hemispheric asymmetries are a prominent but model-dependent feature of mTBI. While they are most evident in lateralized injury paradigms, where they may partly reflect the geometry of injury, multiple lines of evidence point to active interhemispheric differences in plasticity, metabolism, and functional outcome, including contralateral compensatory responses and side-dependent behavioral effects. Notably, hemispheric asymmetries appear to be more consistently detectable in rat models, whereas mouse models yield more variable results, ranging from subtle side-dependent functional effects to symmetric damage patterns. However, given substantial differences in injury models, developmental stages, and experimental design, it remains unclear whether these differences reflect true species-specific characteristics or are primarily driven by methodological factors. Overall, these data highlight that hemispheric asymmetries represent not only a methodological consideration, but also a biologically relevant dimension of mTBI pathophysiology that may influence outcome and recovery trajectories.

### 3.4 Models of seizure and epilepsy

Findings in humans experiencing seizures or epilepsy highlight the role of inherent hemispheric lateralization. For example, patients with mesial temporal lobe epilepsy show altered functional connectivity and reduced interhemispheric asymmetry, with distinct patterns in left- and right-sided epilepsy (Zhao et al., 2022). Also, patients with generalized epileptiform discharges in idiopathic generalized epilepsy demonstrated consistent hemispheric asymmetry without a left-right bias, indicating lateralized network involvement (Pizarro et al., 2025).

Hemispheric asymmetries in experimental seizure models vary widely depending on the species, strain, and induction paradigm. In the audiogenic kindling model of generalized tonic-clonic seizures, Wistar and WAG/Rij rats exhibited consistent individual lateralization of early behavioral seizure manifestations, particularly the direction of preconvulsive running. This lateralization was predictive of the initial ipsilateral cortical involvement, suggesting a lateralized brainstem-to-forebrain seizure propagation. While population-level biases were absent, strain-specific differences emerged: Wistar rats showed fewer cortical seizures when running to the right, indicating enhanced resistance of the right hemisphere to epileptogenesis, whereas WAG/Rij rats with mixed genetic epilepsy showed weak lateralization (Vinogradova and Shatskova, 2012).

In the kainate model of temporal lobe epilepsy, comparisons between spontaneously hypertensive rats (SHRs) and Wistar rats revealed left-right differences in baseline electroencephalography (EEG) oscillations, including increased delta power and reduced gamma activity in SHRs during frontal and parietal cortical recordings. These hemispheric differences correlated with higher seizure susceptibility in SHRs, although in the chronic epileptic phase some oscillatory changes became bilateral (Tchekalarova et al., 2016). Similarly, pentylenetetrazole infusion combined with temporal electrical stimulation in Wistar rats demonstrated ipsilateral activation of the temporal lobe under periodic stimulation, whereas random stimulation reduced interhemispheric differences, highlighting the dynamic influence of circuit synchronization on lateralized seizure recruitment (Mesquita et al., 2011).

Intrinsic functional asymmetry of the nigrostriatal dopamine system has also been linked to lateralized seizure-associated behaviors. In healthy rats, bilaterally recorded pentamethylenetetrazol-activated wave-spike discharges showed amplitude asymmetries between hemispheres (Myslobodsky and Rosen, 1979). Rats consistently rotated in the direction opposite to the hemisphere exhibiting lower amplitude discharges, demonstrating a link between hemispheric excitability and motor imbalance. These findings suggest that baseline asymmetry in dopaminergic circuits can modulate both seizure expression and lateralized motor behavior, emphasizing the importance of pre-existing functional lateralization in seizure susceptibility and behavioral outcomes (Myslobodsky and Rosen, 1979). In a prenatal MAM + THAL Sprague-Dawley rat model (prenatal exposure to chemical agents that disrupt brain development), early postnatal brains exhibited hemispheric asymmetry in which one hemisphere and ventricle were enlarged, among other changes, but these differences normalized in adulthood (Fan et al., 2008). During chemically induced seizures in Wistar rats, elemental analyses revealed hemispheric differences in zinc, magnesium, copper, and sodium levels across cortical regions, showing that seizure-induced biochemical responses can also be lateralized (Yorulmaz et al., 2013).

Not all seizure models produce hemispheric asymmetries. In amygdala kindling paradigms with Wistar rats, no systematic left-right differences were observed for after-discharge thresholds, kindling rate, or D2 receptor mRNA expression, indicating that some forms of epileptogenesis may engage both hemispheres symmetrically (Cain et al., 1989; Gelbard and Applegate, 1994).

Taken together, these studies indicate that hemispheric asymmetries in seizure models are highly model-dependent. Early seizure propagation often displays lateralization, particularly in audiogenic or developmental models, whereas kindling and some pharmacological models may produce largely symmetric responses. Baseline functional asymmetries in motor and dopaminergic circuits can further shape lateralized seizure manifestations, linking intrinsic hemisphere-specific excitability to both electrophysiological and behavioral outcomes. Furthermore, inter-strain differences, e.g., Wistar versus WAG/Rij or SHR, suggest that both genetic background and lateralized network properties influence hemispheric seizure susceptibility.

### 3.5 Models of stroke

Hemispheric asymmetries are also relevant in patients with stroke. For example, right-hemispheric ischemic stroke is associated with higher rates of malignant brain edema and cardiovascular complications than left-hemispheric stroke (Li et al., 2021). Inherent asymmetries in vascular anatomy and blood flow may contribute to stroke lateralization. A larger left internal carotid artery angle, bovine aortic arch anatomy, and reduced right middle cerebral artery peak systolic velocity were identified as predictors of left-sided cardioembolic stroke (Abdelnaby et al., 2023).

In experimental stroke models, asymmetries are evident across multiple rodent paradigms, though they depend strongly on age, strain, and lesion location. One older study found side-dependent functional consequences of hemispheric infarcts: adult rats with right middle cerebral artery ligation exhibited a transient period of hyperactivity accompanied by widespread reductions in cortical and subcortical catecholamines, whereas left-sided infarcts produced minimal behavioral or neurochemical effects despite similar lesion size (Robinson and Coyle, 1980).

Neonatal hypoxia-ischemia (HI) models highlight the influence of age, sex, and brain lateralization. In Wistar rats subjected to unilateral HI using the Levine-Rice model, behavioral and morphological outcomes were both hemisphere- and sex-dependent (Arteni et al., 2010). Right hemisphere HI caused greater reductions in the ipsilateral striatal area, particularly in females, while males showed comparatively larger hippocampal volumes. Functionally, right-HI increased locomotor activity in males and impaired working memory in females, whereas aversive learning deficits occurred in both sexes. These findings indicate that the right hemisphere is intrinsically more vulnerable to neonatal HI and that sex modulates both structural damage and task-specific behavioral deficits (Arteni et al., 2010). In HI-P3 Wistar rats, occlusion of the right or left carotid artery at postnatal day 3 produced early biochemical deficits, with decreased Na^+^/K^+^-ATPase activity predominantly in females and in the right hemisphere, suggesting an initial hemispheric and sex-dependent vulnerability (Sanches et al., 2013b). When these animals were assessed for cognitive and behavioral outcomes in adulthood, females with left-hemisphere injury exhibited the most pronounced spatial memory impairments and greater histological damage, while males and right-hemisphere-injured animals were relatively spared, highlighting a dissociation between early biochemical disruption and long-term functional consequences (Sanches et al., 2013a). A follow-up study comparing HI-P3 and HI-P7 rats demonstrated that the age at injury strongly modulates both behavioral impairments and tissue damage: HI-P7 animals showed broader motor and cognitive deficits and more extensive histological damage, whereas HI-P3 animals displayed subtler impairments, predominantly in left hemisphere-injured females, indicating that developmental stage interacts with hemispheric lateralization and sex to shape the long-term impact of neonatal HI (Sanches et al., 2015).

Transient focal ischemia in adult Wistar rats, induced by intraluminal filament occlusion of the right middle cerebral artery, further illustrates lateralized vascular responses. Time-of-flight MR angiography combined with perfusion- and diffusion-weighted imaging confirmed hemispheric differences in vascular branching and lesion reperfusion dynamics, with some animals exhibiting delayed or incomplete reperfusion in the ipsilateral hemisphere (Besselmann et al., 2001). Complementing this, in C57Bl/6 mice, permanent left middle cerebral artery occlusion produces pronounced hemispheric asymmetry. The injured hemisphere shows larger infarct volume, incomplete vascular perfusion, and increased infiltration of inflammatory cells, while the contralateral side remains largely unaffected. Molecular analysis confirms that inflammatory responses are also hemisphere-specific (Novitzky et al., 2016).

Oligodendrocyte structures also show early hemisphere-dependent responses following focal cerebral ischemia. In 3- and 12-month-old Sv129/B6 wild-type and triple-transgenic Alzheimer-model mice, unilateral middle cerebral artery occlusion elicited enhanced immunoreactivities for myelin basic protein and oligodendrocyte-specific protein predominantly in the ischemic core, with a shell-like decline toward peri-ischemic cortex. These inter-hemispheric differences were consistent across ages and genotypes (Michalski et al., 2018).

Anesthetic and pharmacological manipulations can also modulate hemispheric vulnerability. During left unilateral carotid occlusion and hypotension, EEG recordings in adult rats demonstrated that the earliest amplitude decreases occurred ipsilateral to the occluded artery, with nitrous oxide showing minimal impact on the MAP threshold for EEG asymmetry, suggesting intrinsic hemispheric differences in ischemic susceptibility rather than drug-mediated effects (Zhou et al., 1989).

Individual functional lateralization also modulates recovery after stroke. In adult rats, lesions affecting the dominant hemisphere (identified via pre-testing for forelimb preference) resulted in significantly better recovery of forelimb function compared with lesions in the non-dominant hemisphere, even when overall neurological deficits was similar (Volkov et al., 2011). Forelimb function was monitored over 8 weeks using cylinder, whisker, and swimming tests, highlighting that intrinsic lateralization of motor circuits influences post-stroke recovery trajectories. This finding suggests that hemisphere-specific dominance should be considered when evaluating functional outcomes in preclinical stroke models (Volkov et al., 2011).

Overall, these studies underscore that lateralized stroke outcomes in rodent models depend on species, strain, age, sex, lesion location, and experimental manipulations. Neonatal HI models reveal that early brain injury interacts with hemispheric specialization and sex to produce long-term cognitive and structural asymmetries, whereas adult ischemia models predominantly affect the ipsilateral hemisphere, with minimal contralateral impact, emphasizing the dynamic relationship between lesion side, neurovascular responses, pharmacological interventions, and functional dominance. Considering individual intrinsic hemispheric dominance can improve the interpretation of post-stroke recovery and functional outcomes in preclinical studies.

### 3.6 Models of Alzheimer’s disease

Findings from patients with Alzheimer’s disease (AD) reveal compelling evidence for a role of altered hemispheric asymmetries in disease etiology and progression (Lubben et al., 2021; Ocklenburg et al., 2024). For example, patients with AD show an accelerated reduction in cortical hemispheric asymmetry (Roe et al., 2021). Moreover, an asymmetric tau distribution (evident in 70% of patients) is associated with an earlier disease onset and more severe pathology (Lu et al., 2023). Also, left-right asymmetry in tau pathology is linked to amyloid-beta laterality and differential cognitive decline, independent of interhemispheric connectivity (Anijärv et al., 2025). Behaviorally, a slight increase in right-dementia patients on the meta-analytical level, although the evidence was limited (Mundorf et al., 2025b).

Hemispheric asymmetries have been increasingly documented in transgenic mouse models of AD, revealing region- and age-dependent differences in functional connectivity, cellular activation, and synaptic plasticity. In the AppNL-G-F knock-in model, resting-state fMRI revealed robust interhemispheric hippocampal connectivity across age, which progressively declined in older mice. Notably, these animals exhibited a loss of hippocampal hemispheric asymmetry, suggesting early network-level disruptions that precede extensive amyloid plaque deposition and may serve as sensitive biomarkers of preclinical AD (Morrissey et al., 2022).

Functional imaging further highlights lateralized microglial activation in relation to cognition. In AppNL-G-F mice, TSPO-PET scans demonstrated that elevated microglial activation in the right amygdala-entorhinal-hippocampal complex was associated with better spatial learning in the Morris water maze, whereas the left hemisphere showed no such correlation. This right-lateralized microglial activity suggests a protective or compensatory role of microglia in maintaining hippocampal-dependent cognitive function (Biechele et al., 2021). Extending these observations beyond a single model, studies across multiple amyloid-β mouse models (APP/PS1, PS2APP, APP-SL70, AppNL-G-F, and APPswe) reveal that hemispheric asymmetries in plaque burden are common at the individual level, even though there is no consistent population-wide vulnerable hemisphere (Sacher et al., 2020). Furthermore, asymmetries in amyloid-β deposition correlated with lateralized microglial activation, indicating a neuroinflammatory component to hemisphere-specific plaque accumulation (Sacher et al., 2020). Such individual variability may increase experimental variance and act as a potential confounder in preclinical studies.

Electrophysiological studies corroborate these findings at the network level. Bilateral local field potential recordings in APP/PS1 mice revealed increased gamma-band synchronization between left and right secondary motor cortex compared with wild-type controls, consistent with reduced hemispheric asymmetry in cortical network activity (Chen et al., 2019).

Finally, synaptic-level investigations demonstrate pathway-specific lateralization of plasticity. Optogenetically evoked long-term depression (LTD) in the hippocampus of Wistar and Lister hooded rats revealed that soluble amyloid-β preferentially facilitates LTD in the left hippocampus via mGluR5-dependent mechanisms, whereas LTD in the right hippocampus remained largely unaffected. This asymmetric synaptic vulnerability provides a potential mechanistic explanation for emergent hemispheric functional differences in AD (O’Riordan et al., 2018).

Collectively, these studies indicate that hemispheric asymmetries in AD models manifest across multiple levels, from synapses to networks, and are influenced by regional microglial and oligodendrocyte responses. Importantly, recent evidence in mice shows that the hemisphere with higher amyloid plaque burden varies between individual animals rather than being consistent across the population, highlighting a previously underappreciated source of variability. These findings underscore that both the hemisphere affected and the degree of lateralization should be explicitly considered in experimental design and data interpretation, as ignoring such variability may obscure functional or structural effects in rodent models of AD.

### 3.7 Models of Parkinson’s disease

Evidence from Parkinson’s disease (PD) indicates that altered hemispheric asymmetries play a role in disease etiology and progression, as well (Lubben et al., 2021; Ocklenburg et al., 2024). For example, in PD, motor symptoms show a bias toward the dominant side (van der Hoorn et al., 2012) and left-hemispheric dopaminergic loss is associated with cognitive impairment, whereas right-hemispheric loss is associated with greater motor severity (Fiorenzato et al., 2021). Moreover, right-sided symptom onset is associated with more severe white matter damage than left-sided onset (Zhu et al., 2023).

Hemispheric asymmetries in experimental PD models are prominent in rodent paradigms with unilateral lesions of the nigrostriatal dopamine (DA) system. In adult Sprague-Dawley rats with unilateral 6-hydroxydopamine (6-OHDA) lesions of the medial forebrain bundle, decreased expression of corticotropin releasing hormone and neurotensin mRNAs was observed in the central amygdala and the oval nucleus of the bed nucleus of the stria terminalis ipsilateral to the lesion, whereas enkephalin mRNA levels remained unchanged. These changes were region-specific, sparing the ventral bed nucleus of the stria terminalis, paraventricular hypothalamus, cortex, and hippocampal CA1 region, and indicated that mesostriatal DA depletion selectively modulates neuropeptide signaling in stress- and emotion-related circuits (Day et al., 2002).

Subcortical immune modulation also appears to be lateralized in PD models. Unilateral 6-OHDA lesions of the substantia nigra in mice produced hemisphere-dependent alterations in spleen lymphocyte mitogenesis, with left- versus right-lesioned animals showing slight depression or enhancement of proliferation, respectively, while natural killer cell activity remained unaffected. These findings indicate that central DA asymmetry can exert hemisphere-specific immunomodulatory effects (Neveu et al., 1992).

Metabolic asymmetries are further evident in the pedunculopontine nucleus (PPN) of 6-OHDA-lesioned Sprague-Dawley rats, where autoradiographic measurement of glucose utilization revealed increased ipsilateral PPN activity alongside reduced rCMRglucose in primary motor, sensory, and auditory cortex, and modest decreases in the subthalamic nucleus. These hemisphere-specific metabolic alterations underscore the role of mesopontine tegmental structures in integrating basal ganglia outputs and shaping motor deficits in PD models (Carlson et al., 1999).

Environmental toxins and age interact with hemispheric vulnerability in PD models. In C57Bl6 mice, repeated paraquat exposure induced long-term, predominantly unilateral loss of dopaminergic neurons in the substantia nigra pars compacta (SNc), alongside microglial activation, elevated stress hormones, and delayed inflammatory responses. High-resolution MRI detected modest hemispheric differences in SNc volume and time-dependent enlargement of ventricles, suggesting asymmetric nigrostriatal pathology and persistent neuroinflammatory effects (Dwyer et al., 2021).

Beyond motor circuits, asymmetric dopamine depletion in the nucleus accumbens (NAc) produces side-specific cognitive and affective deficits. Wistar-Han rats with left NAc lesions exhibited increased impulsivity, whereas right NAc lesions impaired the shift from habit-based to goal-directed decision-making. These effects occurred without global motor deficits, highlighting lateralized roles of the NAc in cognitive control and impulsivity (Cunha et al., 2020). Of note, dopamine in the rats’ nucleus accumbens is inherently asymmetrical (Budilin et al., 2008).

At the receptor level, striatal dopamine D1 receptors exhibit lateralized functional properties. In 6-OHDA-lesioned Wistar rats rendered dyskinetic with L-DOPA, left striatal D1 receptors showed higher agonist affinity than right receptors, and D3 receptor activation could normalize left-right asymmetry. These findings indicate that dyskinesia emerges with hemisphere-dependent receptor dynamics rather than purely expression-level changes, highlighting a functional lateralization of striatal neurotransmission (Farré et al., 2015).

Electrophysiological studies support functional lateralization of basal ganglia circuits. In 6-OHDA-lesioned female Sprague-Dawley rats, unilateral right-sided lesions reduced firing rates in ipsilateral SNr neurons while increasing contralateral firing, with concomitant changes in bursting patterns, potentially underlying behavioral asymmetries (Rohlfs et al., 1997). In female Wistar rats, after unilateral 6-OHDA-lesion, methamphetamine induced Fos expression in the striatum and substantia nigra pars reticulata in a hemisphere-specific manner, differentially modulated by D1, D2, and NMDA receptor antagonists, while intrastriatal grafts suppressed rotational behavior but not SNr Fos expression, suggesting that distinct neuronal pathways independently drive lateralized molecular and behavioral responses (Ishida et al., 1998a,b). Together, these studies demonstrate that unilateral dopaminergic lesions produce coordinated yet region- and mechanism-specific hemispheric asymmetries at both the electrophysiological and molecular level.

In addition to classical motor deficits, unilateral dopaminergic lesions also produce asymmetries in sensorimotor orientation, affective processing, and prefrontal cortical functions. Sprague-Dawley rats with unilateral nigrostriatal 6-OHDA lesions display biased orientation to edges in open-field tests (Sullivan et al., 1994), postural akinesia (Johnson et al., 1999; Long-Evans rats), and lateralized basal ganglia metabolism (Kozlowski and Marshall, 1983). Similarly, unilateral lesions of mesocortical or mesolimbic dopamine pathways reveal hemisphere-specific effects on anxiety-like behaviors, rewards perception, and memory consolidation, often with left-sided predominance (Sullivan et al., 2014; Papp et al., 2019; Sprague-Dawley and Wistar, respectively). Although these studies do not always measure classical Parkinsonian motor outcomes, they demonstrate that unilateral DA depletion (commonly used in PD models) produces persistent lateralized changes in sensorimotor, cognitive, and affective circuits. Notably, comparable asymmetries in cognitive and affective domains have also been reported in patients with Parkinson’s disease (Lubben et al., 2021; Steinbach et al., 2021), supporting the translational relevance of these findings. Together, this highlights the broad impact of asymmetric nigrostriatal and mesocorticolimbic dopaminergic dysfunction.

Taken together, these studies demonstrate that unilateral dopaminergic lesions or environmental insults produce widespread hemispheric asymmetries affecting neuropeptide signaling, immune function, neuronal activation, metabolism, and cognition. The direction and magnitude of these effects depend on lesion site, neuronal circuit, receptor dynamics, and environmental or pharmacological manipulations. Understanding these lateralized patterns is critical for interpreting preclinical PD models and for developing interventions that may differentially target affected versus spared hemispheres.

### 3.8 Others

In humans, systematic evidence for the role of inherent hemispheric asymmetries in other neurological conditions remains limited. Studies in glioma suggest that right-hemispheric tumors are associated with higher preoperative anxiety, whereas left-hemispheric tumors are linked to poorer survival outcomes (Zhao et al., 2025). Animal models provide additional insights into the underlying mechanisms.

In rats receiving unilateral ibotenic acid lesions of the ventromedial globus pallidus, which disrupts the primary source of cortical cholinergic innervation, early post-lesion measurements (3 days) revealed significant reductions in cortical glucose utilization in the lesioned hemisphere across multiple cortical areas, whereas the anterior thalamus and dorsal hippocampus remained unaffected. These hemispheric metabolic differences largely normalized by 28–32 days post-lesion. Administration of the muscarinic agonist oxotremorine partially increased glucose utilization in the lesioned frontoparietal cortex but did not eliminate the asymmetry, supporting the idea that cortical metabolic decrements can arise from loss of subcortical input and can be modulated pharmacologically (London et al., 1984).

Similarly, experimental brain tumors induced by ethylnitrosourea in rats demonstrated modest lateralization in blood-to-tissue transport depending on tumor type and vascularity. Autoradiographic measurements of alpha-aminoisobutyric acid transfer revealed that most gliomas exhibited slightly higher unidirectional transfer rates (K) compared to contralateral non-tumorous cortex, with regional increases in larger or more vascularized tumors. In contrast, malignant schwannomas showed highly variable K values, reflecting heterogeneous vascular proliferation, and only limited correlation with specific histological features. These data indicate that hemispheric differences in transport and metabolism can emerge in pathological conditions such as focal lesions or neoplasia, but the extent and persistence of asymmetry are context-dependent (Blasberg et al., 1983).

Taken together, these studies demonstrate that lateralized cortical function is not only influenced by injury or developmental timing, as in stroke or HI models, but can also be affected by subcortical lesions and tumor-induced alterations in metabolism or vascular transport. Hemispheric asymmetries in these models are often transient or modifiable, highlighting the dynamic interplay between structure, connectivity, and functional output.

## 4 Discussion

Hemispheric asymmetries are fundamental to brain function and neurological disease, but preclinical models often focus on unilateral lesions or manipulations, potentially overlooking naturally occurring, individual-specific asymmetries. Genetic and transgenic models reveal that such intrinsic asymmetries exist even in the absence of experimentally imposed damage. For example, in amyloid mouse models of AD, Sacher et al. (2020) showed that fibrillar plaque burden was asymmetric in most animals, yet the side of predominance varied across individuals, demonstrating that lateralized pathology does not necessarily follow a population-level bias. Similarly, behavioral studies of paw preference indicate that most mice (81%) and rats (84%) show a left- or right-side preference, but no population-level bias emerges, emphasizing the importance of inter-individual variability in lateralization (Manns et al., 2021). These individual differences have functional consequences in experimental models. In preclinical stroke paradigms, lesions to the behaviorally dominant hemisphere result in better recovery of forelimb function compared with lesions to the non-dominant hemisphere, even when overall neurological deficit is similar, highlighting that intrinsic lateralization shapes post-stroke outcomes (Volkov et al., 2011).

Across rodent models of neurological disease, including neonatal HI, adult focal ischemia, PD, and AD, subtle, intrinsic hemispheric differences interact with sex, age, and lesion side to determine structural, molecular, and functional outcomes. Neonatal HI models produce hemisphere- and sex-dependent biochemical and cognitive alterations that can shift or reverse during development (Sanches et al., 2013a,b, 2015). Adult ischemia and Parkinsonian models reveal that lesion side, intrinsic lateralization of neural circuits, and dopaminergic and striatal pathways all contribute to asymmetric effects on behavior and network function (Rohlfs et al., 1997; Ishida et al., 1998a,b; Day et al., 2002; Cunha et al., 2020). In AD models, hippocampal connectivity and microglial activation often show subtle asymmetries, reflecting intrinsic differences rather than experimental manipulation alone (Sacher et al., 2020; Biechele et al., 2021; Morrissey et al., 2022). Importantly, not all studies investigating unilateral manipulations consider the potential contribution of hemispheric differences. Our overview highlights that many studies using unilateral manipulations do not consider potential hemispheric differences. Pooling left- and right-sided interventions or not reporting the affected hemisphere may obscure lateralized mechanisms and reduce the explanatory value of experimental findings.

Human data support this principle: although certain cognitive functions show population-level hemispheric trends, such as left-hemispheric dominance for language and right-hemispheric dominance for spatial processing, many individuals exhibit reversed or partially reversed functional lateralization (Genon et al., 2022; Gerrits, 2024; Ocklenburg et al., 2024). Assuming uniform lateralization across all individuals risks misinterpreting disease mechanisms or therapeutic responses. For example, interventions that target a fixed hemisphere, such as unilateral neuromodulation of the left amygdala, may fail or be suboptimal in patients whose functional dominance is reversed or atypical (Mundorf and Ocklenburg, 2025c).

These observations highlight an important translational challenge: neurological animal models often assume a fixed direction of lateralization, potentially overlooking biologically relevant inter-individual variability. Analogous to humans, individual rodents may differ in dominant hemispheres for motor, cognitive, or affective functions. Pre-assessment of hemisphere dominance, using behavioral markers such as paw or forelimb preference (Isparta et al., 2024), combined with imaging or electrophysiology where feasible, can identify the dominant hemisphere in each subject. This allows experimental manipulations to account for inter-individual differences, analyze both hemispheres separately, and, where appropriate, align interventions, such as pharmacology, stimulation, or lesion placement, with dominant versus non-dominant hemispheres.

A final point to consider is the species distribution of the available evidence. Many large-scale studies characterizing hemispheric asymmetry, particularly at the systems and whole-brain level, have been conducted in mice (Elkind et al., 2023; Rivera-Olvera et al., 2024; Silberfeld et al., 2025), whereas a substantial proportion of neurological disease models reviewed here rely on rats. While fundamental aspects of hemispheric lateralization are conserved across species (Güntürkün et al., 2020), this may not extend equally across all levels of organization. In particular, functional and molecular asymmetries may be more likely to vary across species and experimental contexts. Accordingly, it cannot be assumed that findings derived from mice translate directly to rats without further validation. These considerations highlight the importance of cautious interpretation and cross-species validation, especially when drawing conclusions about functional and molecular lateralization in disease models. Collectively, these strategies emphasize the importance of integrating intrinsic hemispheric asymmetry into neurological disease research, thereby enhancing the translational relevance of preclinical findings and reducing the risk of overlooking individual-specific effects on disease progression, recovery, and treatment response.

However, while alterations in hemispheric asymmetry have been documented in several neurological diseases in humans, the availability and extent of clinical evidence remain variable across disease entities. For some conditions, such as AD, converging evidence from patients and experimental models suggests disease-related disruption of lateralized network organization, whereas for other diseases, current evidence is more limited and animal models provide important mechanistic insights that require further clinical validation.

## 5 Limitations

Despite the strengths of this systematic review, several methodological challenges should be acknowledged. Although a comprehensive search strategy was applied across multiple databases, it remains possible that relevant studies were not captured, particularly those using non-standard terminology. The included studies were highly heterogeneous with respect to rodent species, disease models, experimental paradigms, and outcome measures. This heterogeneity reflects the current state of the field and limits direct comparability. In addition, several studies did not distinguish between anatomical subregions, which may have obscured region-specific patterns of hemispheric asymmetry. Furthermore, key methodological details were not consistently reported across studies, such as the animal strain or affected hemisphere, which may affect both the interpretation and reproducibility of findings. Furthermore, approaches to assessing hemispheric asymmetry and lateralization varied considerably between studies, highlighting the lack of standardization in this area and complicating cross-study comparisons. Together, these factors underscore the inherent variability and methodological complexity of animal models used to study neurological diseases.

## 6 Conclusion

In conclusion, hemispheric asymmetry represents both an opportunity and a challenge for animal models of neurological disease. While unilateral lesions and genetic models provide powerful tools to study lateralized pathology, they often overlook intrinsic, individual-specific asymmetries that do not necessarily follow population-level patterns. Incorporating pre-assessment of hemisphere dominance, analyzing both hemispheres separately, and accounting for inter-individual variability, along with factors such as age, sex, lesion location, and developmental stage, can enhance the validity of these models. By integrating these strategies, preclinical research in stroke, epilepsy, AD, and PD can more accurately reflect the complexity of human lateralized pathology and improve translational relevance.
